# Supplementary material for: Evolution of the Jawed Vertebrate (Gnathostomata) Stomach Through Gene Repertoire Loss: Findings from Agastric Species
Source: J Dev Biol. 2025 Aug 5;13(3):27. doi: 10.3390/jdb13030027 (PMC12372087; doi:10.3390/jdb13030027)
Supplement: Supplementary file 1 [file jdb-13-00027-s001.zip › Figure_S1_S2.pdf]

Supplementary Materials:

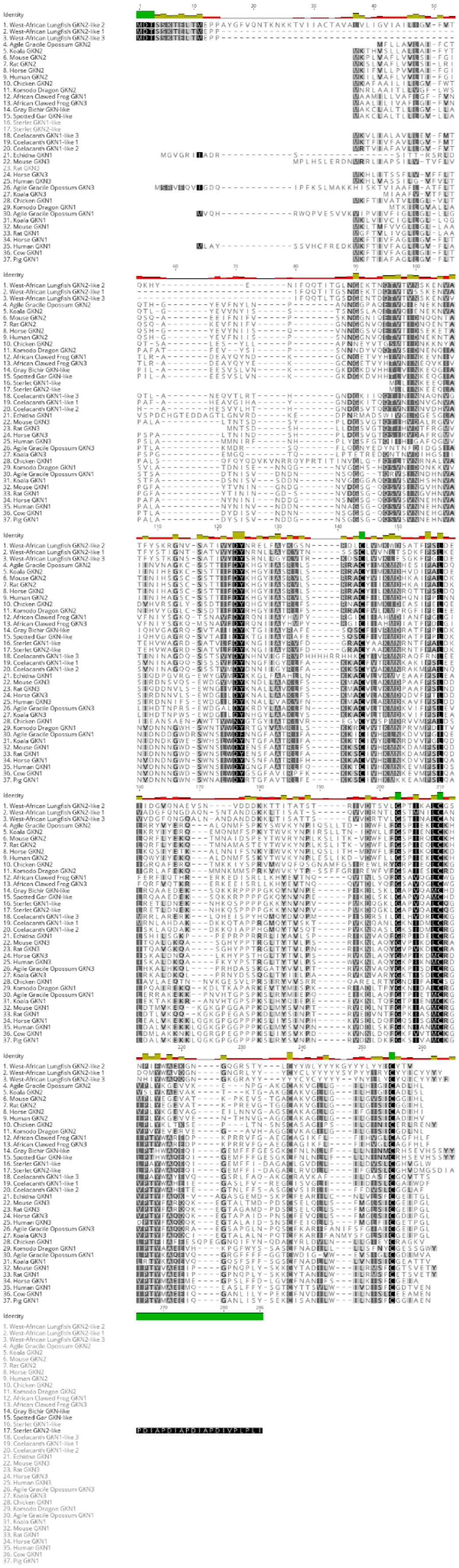

Figure S1: Clustal Omega amino acid alignment of vertebrate gastrokine sequences (GKN1, GKN2, GKN3, GKN-like) with sequence consensus metrics. Dark-shaded boxes indicate 100% sequence identity, whereas lighter-shaded gray boxes indicate lower sequence identity metrics.

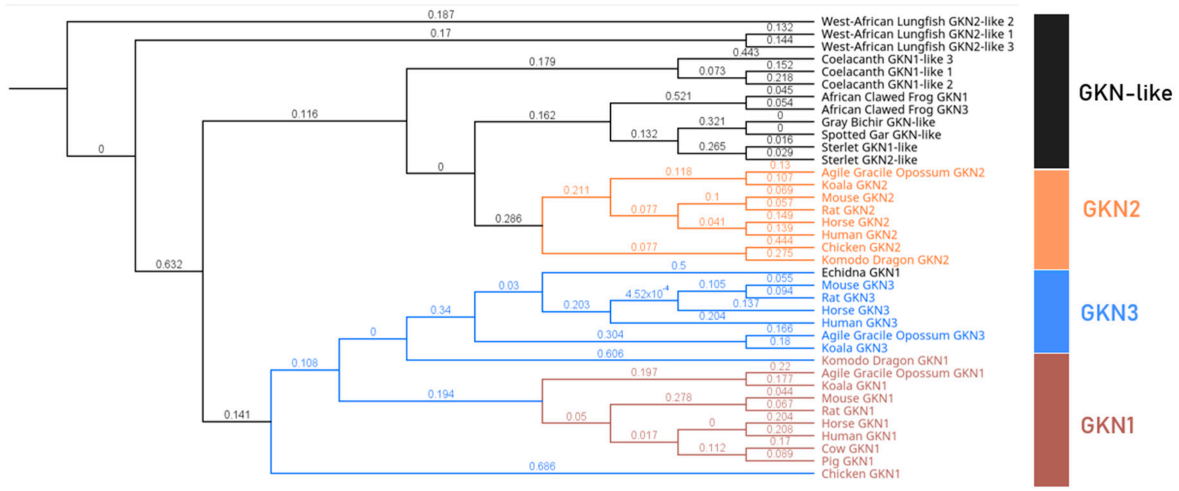

Figure S2: Maximum-likelihood phylogeny of vertebrate gastrokine-like amino acid sequences constructed by the IQ-Tree web server using the JTT + G4 substitution model and 1000 bootstrap replicates. Branch values indicate bootstrap percentage, the scale represents substitutes per site and groupings of gastrokine-like sequences are superimposed on the right.
